# Genome-Wide Identification of the PIN-LIKES (*PILS*) Gene Family in Alfalfa (*Medicago sativa* L.) and Its Expression Analysis Under Abiotic Stresses

**DOI:** 10.3390/cimb48060580

**Published:** 2026-06-01

**Authors:** Xiao Han, Xiaojie Zhang, Rui Wang, Lili Gu, Wenxian Yang, Yiqiang Ren, Zhenwei Ren

**Affiliations:** 1 College of Grassland Science, Xinjiang Agricultural University, Urumqi 830052, China; 2 Xinjiang Key Laboratory of Grassland Resources and Ecology, Urumqi 830052, China; 3 Key Laboratory of Grassland Resources and Ecology of Western Arid Region, Ministry of Education, Urumqi 830052, China

**Keywords:** *Medicago sativa* L., *PILS* gene family, auxin transporter, abiotic stress, gene expression

## Abstract

The *PIN-LIKES* (*PILS*) gene family is crucial for regulating auxin homeostasis and stress adaptation in plants; nevertheless, a comprehensive study on this family in alfalfa (*Medicago sativa*) remains insufficient. This research found 46 *MsPILS* genes within the tetraploid alfalfa genome and categorized them into four subfamilies. The genes are irregularly allocated throughout 16 chromosomes, with tandem duplications acting as a primary catalyst for family expansion. Analysis indicated that all MsPILS proteins contain the conserved Mem_trans domain. The promoter study revealed that *MsPILS* genes had many cis-elements that respond to abiotic stressors and hormones. qRT-PCR research indicated that *MsPILS* genes exhibit variable expression across several tissues and respond to multiple abiotic stressors. Protein–protein interaction (PPI) research revealed PIN3, PIN5, and PIN6 as principal interacting partners of the MsPILS proteins. Subcellular localization studies indicated that MsPILS1c is in the nucleus, plasma membrane, and endoplasmic reticulum (ER). This research offers significant genetic resources and a theoretical framework for elucidating the activities of *PILS* genes and for molecular breeding aimed at improving stress tolerance in alfalfa.

## 1. Introduction

Auxin plays a critical role in regulating diverse aspects of plant growth and development throughout the life cycle [1,2], as well as mediating responses to environmental stresses [3]. To date, four major families of auxin transporters have been identified: the PIN-FORMED (PIN) family [4], the AUXIN1/LIKE-AUX1 (AUX/LAX) family, the ATP-binding cassette subfamily B (ABCB) family [5], and the PILS family [6]. Polar auxin transport mediated by PIN efflux carriers plays a crucial role in auxin distribution [7,8,9]. PILS proteins represent a newly discovered class of auxin transporters that contribute to auxin polar transport [10].

The PILS auxin transporter family was recognized due to its resemblance to PIN proteins and is defined by a conserved domain featuring two transmembrane helices and a brief central hydrophilic loop [6]. PILS proteins are thought to have developed separately from the PIN family and are extensively distributed among green plants, including Chlorophyta [11]. PILS proteins govern intracellular auxin levels in the endoplasmic reticulum, therefore influencing the accessibility of auxin for nuclear signaling. The activity of PILS affects endogenous levels of indole-3-acetic acid (IAA), potentially via intracellular accumulation and metabolism [12]. Endoplasmic reticulum stress modulates PILS protein degradation through the unfolded protein response pathway in a tissue-specific manner, connecting auxin signaling to adaptive plant growth under stress [13]. Research has established that PILS proteins perform various functions: they restrict the pace of nuclear auxin signaling, assimilate external stimuli like light and temperature into auxin-mediated growth processes [14], and govern de novo organogenesis along with the growth rates of roots and shoots [6,15]. PILS proteins, akin to classical PIN proteins, can regulate divergent growth responses in organs; however, their methods differ. PILS proteins do so mainly by establishing spatially localized auxin signaling minima, while the phytochrome B (phyB)-dependent light signaling pathway directly governs. The *AtPILS* gene regulates plant growth transitions by enabling light to modulate nuclear auxin signaling responses [14]. The regulation of PILS protein levels by auxin establishes a feedback loop essential for sustaining cellular homeostasis in auxin signaling output [16]. The auxin response facilitated by *PILS6b* is transcriptionally governed by *OsSPL14*, which is upstream of both *PILS6b* and *OsPIN1b*, therefore influences axillary bud outgrowth [17]. In *Chrysanthemum seticuspe*, *CsPIN2* and *CsPILS6* modulate root stress responses in an ABA-dependent manner to improve drought resistance [18]. *HbPILS6* exhibits responsiveness to various stressors, indicating its function in connecting auxin-mediated development to environmental adaptation [19]. *LiPIN3a* and *LiPILS5b*/*6a*/*6b*/*6c* demonstrated elevated expression levels in salt-tolerant genotypes compared to salt-sensitive ones under salt stress, indicating their possible function as candidate genes in the salt stress response [20]. The findings indicate that PILS family members may contribute to improving plant stress tolerance.

Alfalfa, or lucerne, is a perennial herbaceous legume grown globally. Morphologically, it varies in height from 0.3 to 1 m, featuring serrated leaf margins and predominantly purple flowers [21]. Alfalfa is extensively cultivated in arid regions, principally because of its remarkable environmental resilience and superior nutritional value [22]. Moreover, extensive alfalfa cultivation enhances climatic conditions and the ecological environment [23]. Alfalfa, a cool-season perennial legume, serves as pasture, biofuel feedstock, and soil enhancement. Due to its capacity to absorb nitrogen, sequester carbon, and improve soil structure, alfalfa demonstrates considerable potential for agricultural systems and sustainable development. While historically regarded as moderately salt-tolerant, contemporary alfalfa cultivars exhibit distinct salt-tolerance mechanisms, establishing their suitability as a principal crop for saline-alkali soils [24].

Research is still limited, particularly on perennial fodder crops, despite the identification and characterization of PILS genes in several plant species, such as Areca catechu [25], pineapple [26], tomato [27], rice [28], and maize [29]. Alfalfa, a perennial forage legume grown globally, sometimes encounters substantial obstacles from abiotic factors, including drought and salinity, which limit its growth, development, and productivity [30]. PILS proteins, located in the endoplasmic reticulum, regulate intracellular auxin homeostasis and are essential for plant stress adaptation by integrating endoplasmic reticulum stress signals with auxin signaling [13,14,15]. This suggests that the *PILS* family in alfalfa may have developed specialized roles in synchronizing developmental processes with stress responses. Nonetheless, a comprehensive genome-wide identification of the *PILS* family in alfalfa has yet to be performed, and its expression patterns across various tissues, in response to hormonal stimuli, and under abiotic stress conditions remain significantly under-investigated.

Here, we conducted a genome-wide identification of the *PILS* gene family in alfalfa utilizing bioinformatics methodologies. We conducted a systematic analysis of their evolutionary relationships, chromosomal positions, promoter cis-elements, and syntenic linkages. Additionally, we examined their transcriptional response patterns across multiple organs, in reaction to hormone therapies, and under diverse abiotic conditions. The subcellular location of a crucial family member was experimentally confirmed. These findings present a thorough examination of the *PILS* gene family in alfalfa, elucidating their possible functions in growth, development, and stress adaptation while establishing a basis for the genetic enhancement of this significant fodder crop.

## 2. Materials and Methods

### 2.1. Identification of the Alfalfa PILS Gene Family and Analysis of Physicochemical Properties of Proteins

The whole-genome file of *Medicago sativa* L. cv. ‘Xinjiangdaye’ (*M. sativa*) and its corresponding GFF file were downloaded from the alfalfa genome website (https://figshare.com/projects/whole_genome_sequencing_and_assembly_of_Medicago_sativa/66380, accessed on 7 May 2024) [31]. The whole-genome and GFF files of *Arabidopsis thaliana* (*A. thaliana*) were retrieved from Ensembl Plants (https://plants.ensembl.org/index.html, accessed on 7 May 2024).

After confirming all members and gene designations of the *PILS* family in *A. thaliana* via published literature, the corresponding protein files were acquired from TAIR (https://www.arabidopsis.org/, accessed on 10 May 2024). The hidden Markov model (HMM) profile corresponding to the *PILS* family (PF03457) was obtained from the Pfam database.

Using TBtools software (version 2.476) [32], an initial set of 48 genes was identified in the alfalfa genome through the combined application of BLAST alignment and Pfam searches. Subsequent validation was performed using the InterPro database (https://www.ebi.ac.uk/interpro/search/sequence/, accessed on 10 May 2024), which led to the exclusion of two genes lacking the Mem_trans domain, ultimately resulting in a total of 46 confirmed *MsPILS* family genes.

The physicochemical properties of the MsPILS proteins were evaluated using the ExPASy ProtParam server (https://web.expasy.org/protparam/, accessed on 10 June 2024). Meanwhile, the subcellular localization of these proteins was predicted with the WoLF PSORT online tool (https://wolfpsort.hgc.jp/, accessed on 10 June 2024).

### 2.2. Chromosomal Distribution of MsPILS Genes

Chromosomal location data of *MsPILS* genes were retrieved from the GFF file of the *M. sativa* genome. The mapping onto chromosomes was carried out and subsequently visualized using MG2C (http://mg2c.iask.in/mg2c_v2.0/, accessed on 27 July 2025).

### 2.3. Phylogenetic Analysis of the PILS Family

Genome and annotation files for *Medicago truncatula* (*M. truncatula*) and *Oryza sativa* ssp. Japonica (*O. sativa*) were retrieved from the Ensembl Plants database (https://plants.ensembl.org/index.html, accessed on 31 August 2024). A phylogenetic tree was generated using MEGA11 software (version 11.0.13) via the neighbor-joining (NJ) method with 1000 bootstrap replicates, based on the PILS protein sequences derived from *M. sativa*, *A. thaliana*, and *O. sativa*. Multiple sequence alignment of alfalfa PILS proteins was performed with DNAMAN9. The resulting phylogenetic tree was further optimized using the online tool iTOL (https://itol.embl.de/, accessed on 12 August 2025) [33].

### 2.4. Analysis of MsPILS Gene Structure, Conserved Motifs, and Domains

Analysis of conserved motifs in MsPILS proteins was performed using the online MEME suite (https://meme-suite.org/meme/tools/meme, accessed on 21 August 2024), with the number of motifs set to 10. Prediction of conserved domains was carried out via the NCBI Conserved Domain Database (https://www.ncbi.nlm.nih.gov/cdd/, accessed on 21 August 2024). Gene structure information, specifically the exon-intron organization of *MsPILS* genes, was extracted from the GFF annotation file of the alfalfa genome. The TBtools software was employed to visualize the results from the above analyses.

### 2.5. Prediction of Protein Secondary and Tertiary Structures

Protein secondary structures, including *α*-helices, *β*-sheets, *β*-turns, and random coils, were predicted using SOPMA (https://npsa.lyon.inserm.fr/cgi-bin/npsa_automat.pl?page=/NPSA/npsa_sopma.html, accessed on 24 August 2024), while tertiary structures were modeled using SWISS-MODEL (https://swissmodel.expasy.org/, accessed on 24 August 2024).

### 2.6. Protein–Protein Interaction Analysis of the PILS Gene Family in M. sativa

Protein–protein interactions (PPIs) were predicted using the STRING database (https://cn.string-db.org/, accessed on 26 September 2025). The analysis was conducted with a minimum interaction score of 0.70. The maximum number of displayed interactors was set to 10.

### 2.7. Analysis of Promoter Cis-Acting Elements

Prediction of cis-regulatory elements within the 2000 bp promoter regions upstream of the transcription start sites of the 46 *MsPILS* genes was performed using the PlantCARE database (http://bioinformatics.psb.ugent.be/webtools/plantcare/html/, accessed on 2 September 2025) [34]. Visualization of the results was conducted with TBtools software.

### 2.8. Synteny Analysis of MsPILS Genes

Whole-genome and annotation files for *M. truncatula*, *O. sativa*, and *A. thaliana* were downloaded from Ensembl Plants. Using the “One Step MCScanX—Super Fast” plugin in TBtools, intra-species and inter-species synteny analyses were performed. Using TBtools, we calculated the number of synonymous substitutions per synonymous site (Ka) (Table S1), the number of nonsynonymous substitutions per nonsynonymous site (Ks), and the *p*-value from Fisher’s exact test of neutrality. Typically, a Ka/Ks ratio <1, =1, or >1 indicates purifying selection, neutral selection, or positive selection, respectively [35].

### 2.9. Plant Materials, Growth, and Stress Conditions

Seeds of *Medicago sativa* L. cv. ‘Xinjiangdaye’ were obtained from the College of Grassland Science, Xinjiang Agricultural University. The seeds were sterilized and then incubated in Petri dishes at 25 °C. After three days of incubation, the seedlings were transferred to hydroponic culture containing 1/2 Hoagland nutrient solution under controlled conditions: 25 °C, a 16 h light/8 h dark photoperiod, and 60% relative humidity. The nutrient solution was renewed every 3–4 days. After four weeks of growth, seedlings were exposed to cold stress (4 °C) or to nutrient solutions supplemented with 15% PEG6000, 200 mmol/L NaCl, or 100 μmol/L each of abscisic acid (ABA), salicylic acid (SA), and gibberellin (GA).

Seedlings grown in a normal nutrient solution served as the control. Fresh leaf samples of alfalfa were collected from both control and treatment groups at six time points: 0, 3, 6, 12, 24, and 48 h. For tissue-specific expression analysis, alfalfa plants were grown outdoors. Roots, stems, leaves, flower buds, and flowers were collected at the flowering stage, and pods were collected at the podding stage. All samples were immediately frozen in liquid nitrogen and stored at −80 °C for subsequent RNA extraction.

### 2.10. Total RNA Extraction and qRT-PCR Analysis

Primers employed in this study were designed using Oligo 7 software (Table S2). Total RNA was isolated from all samples with the TriQuick reagent (Solarbio, Beijing, China). Following that, cDNA was synthesized according to the manufacturer’s protocol using the NovoScript^®^ Plus All-in-one 1st Strand cDNA Synthesis SuperMix (gDNA Purge) kit (NovoProtein, Suzhou, China). The reaction mixture comprised 10 μL of 2× NovoScript^®^ Plus 1st Strand cDNA Synthesis SuperMix, 0.1 ng–1 μg of total RNA, 1 μL of gDNA Purge, and RNase-free water to a final volume of 20 μL. The thermal profile was set as follows: incubation at 50 °C for 15 min, heating at 85 °C for 5 s, followed by holding at 4 °C. The synthesized cDNA was kept at −20 °C.

Quantitative real-time PCR (qRT-PCR) analysis was conducted using the AGS9900 Real-Time PCR System (Hangzhou, China). The qRT-PCR reactions were performed with the NovoStart^®^ Fast SYBR qPCR SuperMix kit (NovoProtein). Each reaction mixture contained 10 μL of 2× NovoStart^®^ Fast SYBR qPCR SuperMix, 1 μL of cDNA, 1 μL of forward primer, 1 μL of reverse primer, and 7 μL of RNase-free water. The thermal cycling program consisted of an initial denaturation at 95 °C for 1 min, followed by 40 cycles of denaturation at 95 °C for 20 s and annealing/extension at 60 °C for 1 min. Each sample was run in three technical replicates. The *MsActin* gene (AA660796) of *M. sativa* served as an internal reference, and the relative expression levels of genes were determined via the 2^−ΔΔCt^ method [36]. For tissue-specific expression analysis, the expression abundance of selected members of the PILS gene family in alfalfa was assessed in roots, stems, leaves, flower buds, flowers, and pods, with root tissue serving as the control [37].

### 2.11. Vector Construction and Subcellular Localization

Using the primers for subcellular localization (Table S2), the complete coding sequence (CDS) of MsPILS1c (excluding the stop codon) was amplified and then inserted into the *SacI* and *XbaI* restriction sites of pCAMBIA2300-*GFP*. The resulting recombinant construct and the empty pCAMBIA2300-*GFP* vector (control), together with an ER marker (mCherry-ER-RK) obtained from Shanxi Aiyouji Biotechnology Co., Ltd. (Yangling, China), were separately transformed into *Agrobacterium tumefaciens* strain GV3101. Positive transformants were selected and cultured overnight. Overnight-cultured *Agrobacterium* cells were collected and resuspended in infiltration buffer, and the suspension was adjusted to a final OD_600_ of 0.4. The third and fourth leaves from the top of tobacco plants were selected for agroinfiltration. After injection, the treated plants were placed in a culture room in darkness for 24 h, then cultured under conventional light conditions. Two days after infiltration, the infected leaf tissues were excised. The leaf epidermis was stripped and mounted for observation of subcellular localization under a confocal laser scanning microscope using an FV10-ASW system (Olympus Corporation, Tokyo, Japan).

## 3. Results

### 3.1. Identification and Physicochemical Properties of MsPILS Genes

After eliminating repetitive sequences and validating the conserved domain, 46 non-redundant genes expressing the Mem_trans domain (PF03457) were discovered in the alfalfa genome. They were classified as MsPILS1a through MsPILS16d according to their physical arrangement on chromosomes (Table S3). We conducted a systematic investigation of the physicochemical properties of the 46 MsPILS proteins. The length of the protein varied from 131 to 464 residues. The predicted molecular weight varied from 13.7 to 51.9 kDa, and the isoelectric point exhibited significant variation among the proteins. Theoretical pI values varied between 5.10 and 10.52. A total of 23 members exhibited acidity (pI < 7), 22 displayed basicity (pI > 7), and one was neutral (pI = 7). The overall average of the hydropathy index was positive for all members, varying from 0.375 to 1.266, signifying a hydrophobic characteristic. Predictions of subcellular localization indicated varied compartmentalization: 39 proteins were anticipated to localize to the plasma membrane, 4 to the vacuole, and 3 to the endoplasmic reticulum. This differential localization suggests possible functional specializations within specific cellular compartments. These foundational investigations collectively offer essential insights into the functional divergence among the MsPILS family members.

### 3.2. Chromosomal Localization of the MsPILSs Gene

**Figure 1:**
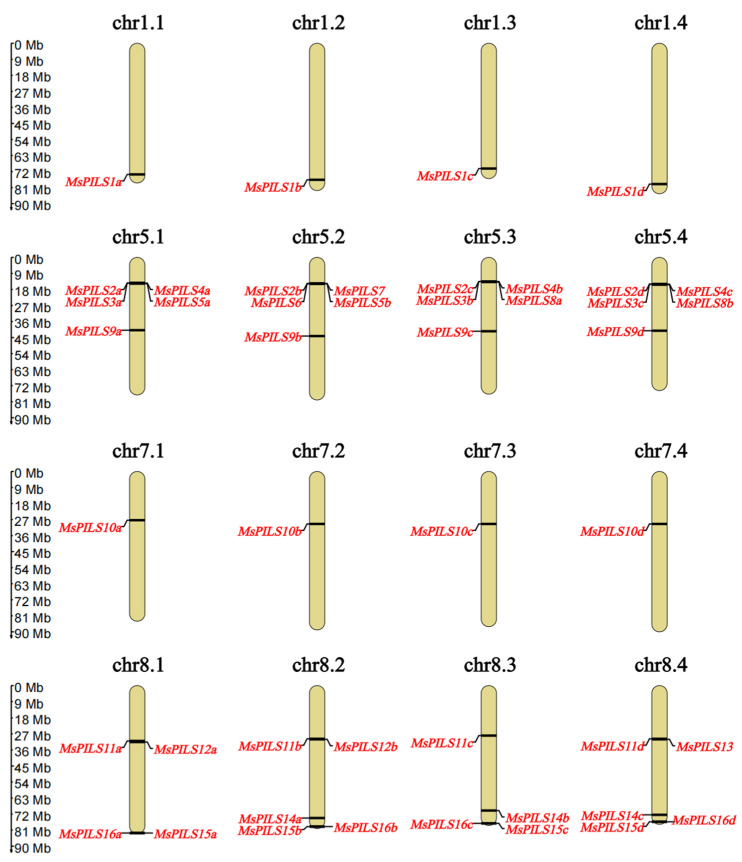
Chromosomal distribution map of the *PILS* gene family in *M. sativa*.

The chromosomal positions of all identified *MsPILS* genes were delineated to examine potential paralogous interactions (Figure 1). The map comprises four homologous chromosomal pairs: Chr1.1–Chr1.4, Chr5.1–Chr5.4, Chr7.1–Chr7.4, and Chr8.1–Chr8.3, among others. The 46 *MsPILS* genes are irregularly allocated across 16 chromosomes. Chromosomes 5 and 8 contain the highest number of genes, with Chr5.1, Chr5.2, Chr5.3, Chr5.4, Chr8.2, and Chr8.4 each comprising five members, while Chr8.1 and Chr8.3 each include four *MsPILS* genes. Additionally, Chr1.1, Chr1.2, Chr1.3, Chr1.4, Chr7.1, Chr7.2, Chr7.3, and Chr7.4 each comprise a singular member.

### 3.3. Phylogenetic Analysis of the MsPILSs Gene Family

**Figure 2:**
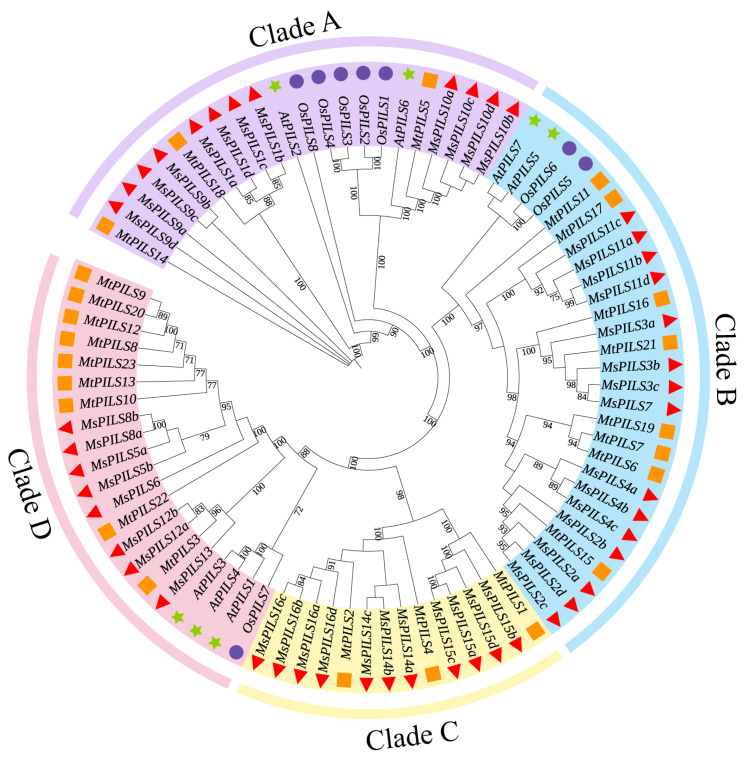
Phylogenetic analysis of *PILS* family members, which are a group of proteins involved in plant growth and development, from *M. truncatula*, *O. sativa*, *A. thaliana*, and *M. sativa*. Shapes denote gene families: orange squares, *MtPILS*; purple circles, *OsPILS*; green pentagons, *AtPILS*; red triangles, *MsPILS*.

A phylogenetic tree was created to examine the evolutionary relationships among MsPILS proteins, utilizing 8 *O. sativa*, 7 *A. thaliana*, 46 *M. sativa*, and 23 *M. truncatula* PILS proteins found in this investigation (Figure 2, Table S4). The 84 *PILS* genes were categorized into four principal clades. Clade A consisted of 22 *PILS* genes. Clade B, comprising 32% of the total *PILS* genes, encompassed 2 *A. thaliana* genes, 2 *O. sativa* genes, 15 *M. sativa* genes, and 8 *M. truncatula* genes. Clade C comprised 11 *M. sativa* genes and 3 *M. truncatula* genes. Clade D, comprising 21 members, represented 25% of the total *PILS* genes. This clade included three *A. thaliana* genes, one *O. sativa* gene, eight *M. sativa* genes, and nine *M. truncatula* genes. The phylogenetic study indicated that PILS proteins from alfalfa and *M. truncatula* exhibited a closer relationship to one another than to those from *O. sativa* and *A. thaliana*.

### 3.4. Analysis of Gene Structure, Conserved Domains, and Motifs of MsPILSs Gene Family Members

**Figure 3:**
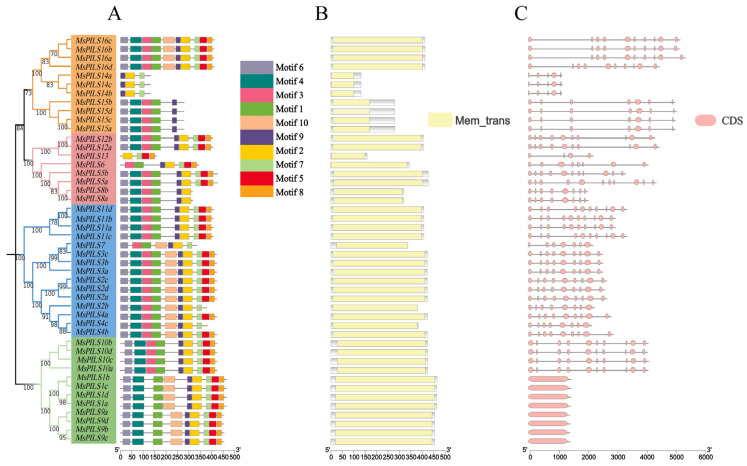
Sequence structure analysis of MsPILSs in alfalfa. (**A**) Motif distribution of MsPILS proteins. Different colors indicate different motifs (1–10). (**B**) Domain distribution of MsPILS proteins. (**C**) Exon–intron structure of *MsPILSs*.

We conducted a conserved motif analysis utilizing the online MEME service to examine motif diversity among MsPILS proteins (Figure 3A). The research revealed 10 unique conserved motifs, labeled motifs 1 through 10. Motifs 1, 2, and 9 were uniformly seen in all MsPILS proteins. Specifically, 12 proteins—MsPILS2a, MsPILS2c, MsPILS2d, MsPILS3a, MsPILS3b, MsPILS3c, MsPILS4a, MsPILS4b, MsPILS16a, MsPILS16b, MsPILS16c, and MsPILS16d—exhibited all 10 motifs. MsPILS14a, MsPILS14b, and MsPILS14c had the lowest number of motifs, including three. MsPILS13 was the sole protein that included motifs 2, 5, 7, and 8. Each position inside the conserved motifs is represented by the relevant amino acid’s conventional single-letter code (Figure S1). Additionally, all MsPILS proteins possessed the Mem_trans domain (Figure 3B). The multiple sequence alignment further validated the presence of the Mem_trans domain in all MsPILS proteins (Figure S2). The findings demonstrate that the majority of MsPILS proteins possess conserved motifs at both termini of their sequences.

The exon–intron architectures were studied to thoroughly elucidate the functional variety of *MsPILS* genes. Analysis of gene structure disclosed varied patterns among distinct *MsPILS* genes. *MsPILS5a* possessed the greatest quantity of exons (11), whereas *MsPILS1a*, *MsPILS1b*, *MsPILS1c*, *MsPILS1d*, *MsPILS9a*, *MsPILS9b*, *MsPILS9c*, and *MsPILS9d* exhibited the least amount of exons (Figure 3C).

### 3.5. Analysis of Protein Secondary and Tertiary Structures

The secondary structure analysis of PILS-encoded proteins in *M. sativa* indicated that this protein family predominantly comprises *α*-helices, extended strands, and random coils, with no *β*-turns identified (Table S5). Among all analyzed PILS proteins, *α*-helices constituted the primary component, varying from 36.90% to 51.90%. Extended strands exhibited relative stability, ranging from 10.13% to 15.19%, whereas random coils constituted 32.91% to 50.00%. The prediction of tertiary structure indicated that *α*-helices function as the structural core, whereas extended strands and random coils enhance a stable three-dimensional conformation (Figure S3). The helix number, helix length, and distribution of flexible regions exhibited considerable variation among subfamilies, but homologous copies within the same subfamily demonstrated good conservation, indicating co-evolution of structure and function.

### 3.6. Protein Interaction Analysis of MsPILS

**Figure 4:**
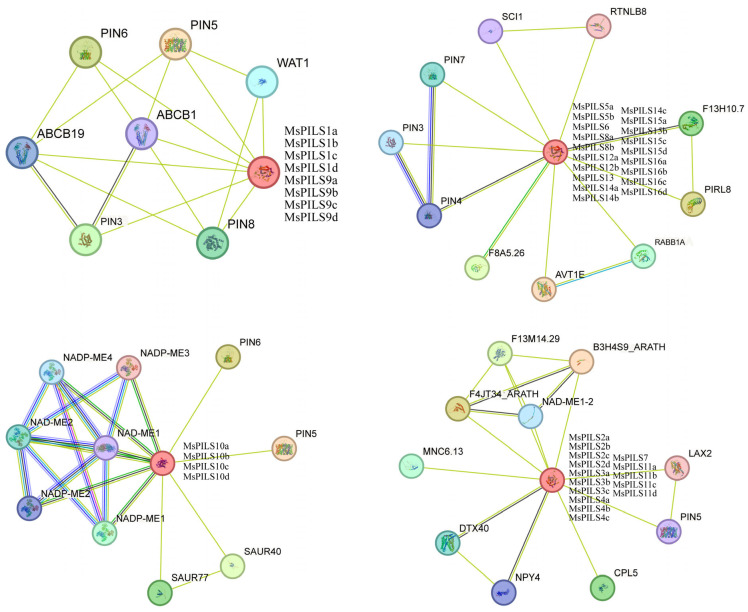
Predicted regulatory network of MsPILS and its interacting proteins. Interaction evidence is distinguished by color coding: curated database records are visualized in sky blue, experimentally validated interactions in purple, gene co-occurrence in dark blue, text mining results in green, co-expression in black, and protein homology in light blue.

Utilizing their *A. thaliana* homologs, PPI networks for MsPILS proteins were constructed, indicating that these homologs exhibit analogous interaction patterns (Figure 4). Specifically, MsPILS1a-MsPILS1d and MsPILS9a-MsPILS9d each engaged with seven proteins, while MsPILS10a-MsPILS10d connected with ten proteins. PIN3, PIN5, and PIN6—essential constituents of the PIN family of auxin efflux carriers vital for polar auxin transport and signaling—were consistently recognized within these interaction networks. This study shows that PILS proteins may be involved in auxin production or in sorting inside the ER and could influence plant growth, development, and tropic responses via manipulation of PIN proteins. Moreover, MsPILS10a-MsPILS10d engaged with several NADP-malic enzyme (NADP-ME) proteins, indicating a possible involvement in energy metabolism or stress adaptation, thereby influencing the control of stress resistance and growth rates in *M. sativa*.

### 3.7. Analysis of Cis-Acting Elements in MsPILSs

**Figure 5:**
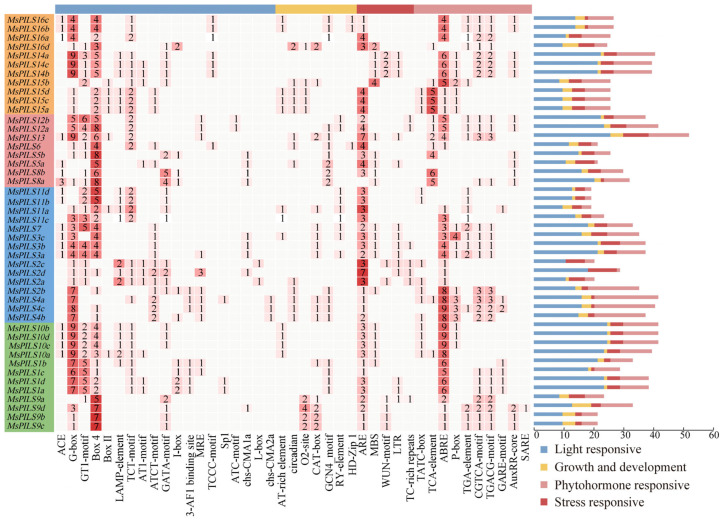
Analysis of cis-acting elements in the promoter regions of the *PILS* family genes in *M. sativa*. The accompanying bar chart presents the number of cis-acting elements assigned to each functional class.

To identify potential cis-acting elements involved in the transcriptional regulation of *MsPILS*, we utilized the PlantCARE public database to functionally annotate all cis-acting elements within the 2.0 kb promoter regions upstream of the ATG start codon of each *MsPILS* gene [34]. A total of 41 major putative cis-acting elements were identified; all of these elements have counterparts in published studies, and their functional classifications were determined with reference to relevant literature [38,39,40,41] (Table S6). They were categorized into four functional groups: light-responsive elements, growth and development-related elements, stress-responsive elements, and hormone-responsive elements [42] (Figure 5, Table S7). Light-responsive features were the most prevalent, encompassing standard G-box, Box 4, and GT1 patterns. The elements associated with growth and development comprised eight types: AT-rich element, circadian, O2-site, CAT-box, GCN4_motif, RY-element, and HD-Zip 1. Five types of stress-responsive elements were identified: ARE, MBS, WUN-motif, LTR [43], and TC-rich repeats. Phytohormone response elements comprise 10 types: TATC-box, TCA-element, ABRE, P-box, TGA-element, CGTCA-motif, TGACG-motif, GARE-motif, AuxRR-core, and SARE.

### 3.8. Collinearity Analysis of MsPILS Gene Family Members

**Figure 6:**
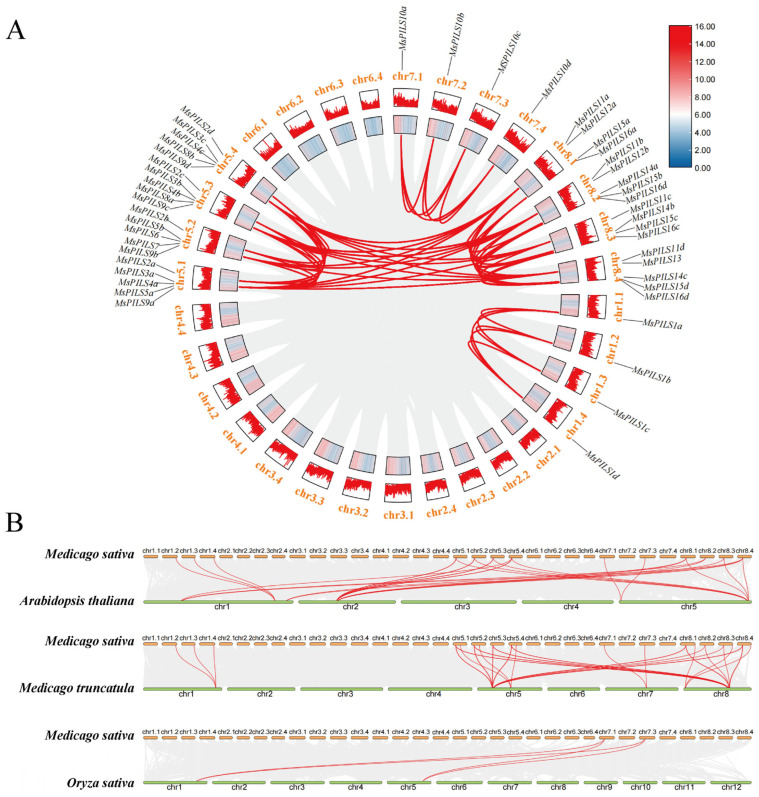
Synteny analysis of *PILS* genes in *M. sativa*. (**A**) Synteny analysis within *M. sativa*. (**B**) Synteny analysis between *M. sativa* and *A. thaliana*, *M. truncatula*, and *O. sativa*. Red lines denote the collinear gene pairs.

Gene duplication is essential for the creation of new genes and functions, with segmental and tandem duplications serving as primary catalysts for the growth of gene families. Our analysis identified 29 segmental duplication events within the 46 *MsPILS* family members (Figure 6A). Synteny maps were developed among *M. sativa*, *A. thaliana*, *M. truncatula*, and *O. sativa* to depict the evolutionary relationships of the *PILS* gene family. We detected 24 homologous gene pairs between *M. sativa* and *A. thaliana*, 24 couples between *M. sativa* and *M. truncatula*, and 4 pairs between *M. sativa* and *O. sativa*. The findings indicated that the 46 *MsPILS* genes displayed synteny with genes in *A. thaliana*, *M. truncatula*, and *O. sativa*, signifying a largely conserved distribution of *PILS* genes throughout these four plant species (Figure 6B). This outcome indicates that these genes emerged prior to the separation of monocots and dicots and have remained significantly preserved.

### 3.9. Expression of MsPILS Genes Under Abiotic Stresses

**Figure 7:**
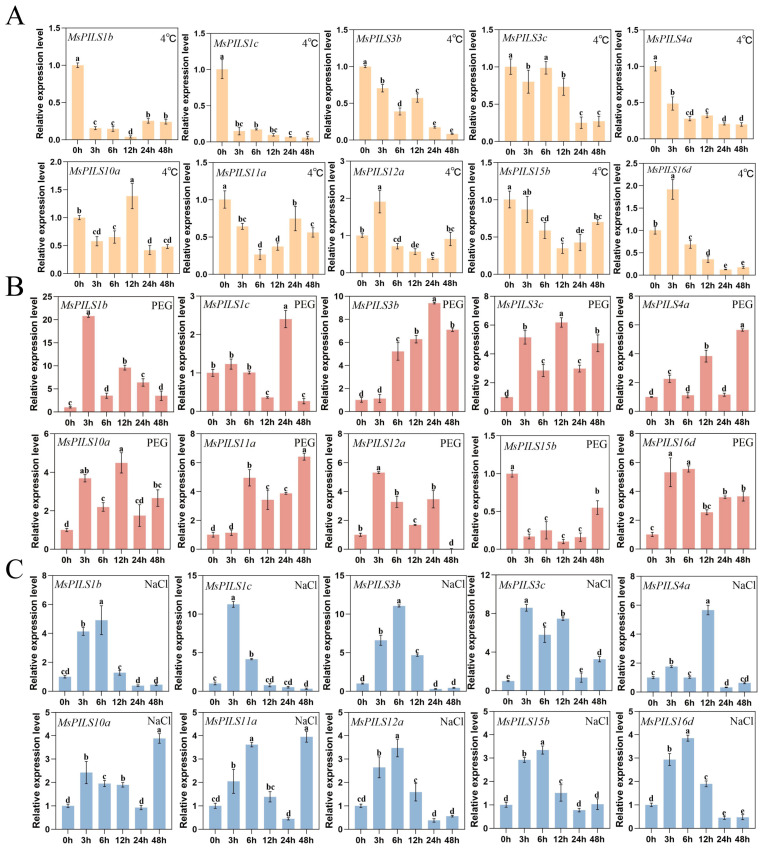
Expression patterns of *MsPILS* genes under cold (**A**), drought (**B**), and salt (**C**) stress (mean ± SD, *n* = 3). One-way ANOVA and Duncan’s multiple range test (*p* < 0.05). Different letters indicate significant differences.

The expression profiles of *MsPILS* genes in response to cold stress were illustrated (Figure 7A). With the exception of *MsPILS10a*, *MsPILS12a*, and *MsPILS16d*, which exhibited upregulation during cold stress, the remaining genes showed downregulation relative to the control (0 h). *MsPILS12a and MsPILS16d* attained their peak relative expression levels at 3 h, subsequently experiencing a decline. *MsPILS10a* reached its zenith at 12 h. *MsPILS16d* demonstrated a consistent decrease in expression starting at 3 h.

The expression patterns of *MsPILSs* during PEG-induced drought stress are illustrated (Figure 7B). PEG treatment markedly influenced the expression of the *PILS* gene family. *MsPILS1b* attained its peak relative expression at 3 h, around 20 times that of the control. The expression of *MsPILS12a* was at its nadir at 48 h, almost 32 times lower than that of the control. *MsPILS15b* exhibited a continuous decline in relative expression from the initiation of treatment to 48 h. Furthermore, nearly all genes demonstrated a pattern of initial elevation, succeeded by a decline, followed by a further increase and a final reduction post-PEG treatment.

The expression profiles of *MsPILSs* during NaCl-induced salinity stress were illustrated (Figure 7C). All identified *MsPILS* genes exhibited significant alterations in response to NaCl stress. The upregulation of 10 genes was predominantly noted during 3 h, 6 h, and 12 h. Reduced relative expression was predominantly noted at 24 h and 48 h. *MsPILS1c* attained its peak relative expression at 3 h, almost 11 times that of the control. Furthermore, *MsPILS3b*, *MsPILS3c*, and *MsPILS16d* exhibited significant alterations from 3 h to 12 h.

### 3.10. Expression of MsPILS Genes Under Phytohormone Treatments

**Figure 8:**
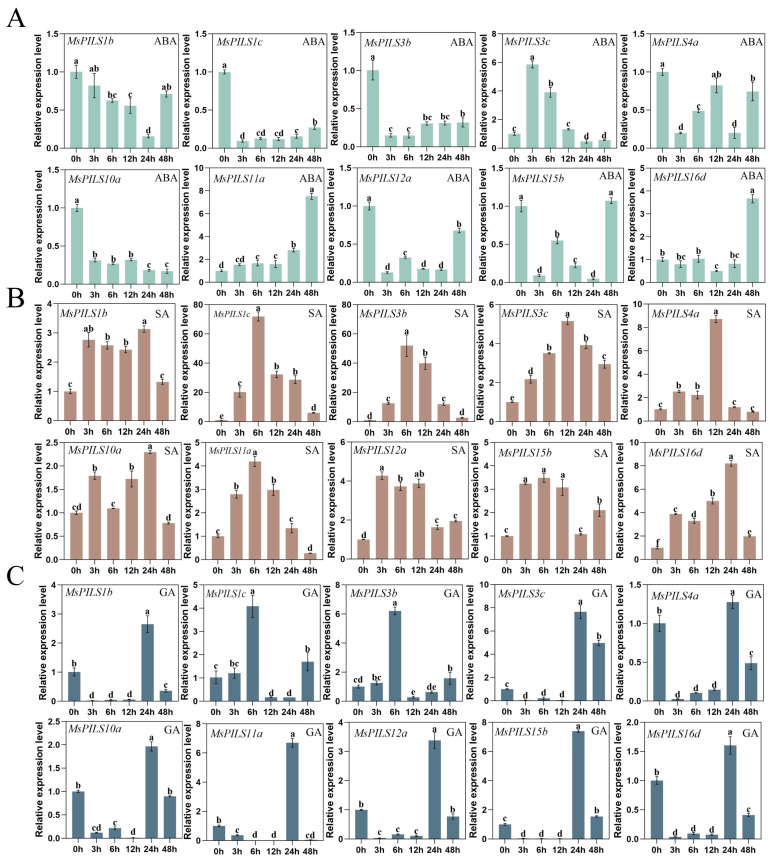
Expression levels of *MsPILS* genes under ABA (**A**), SA (**B**), and GA (**C**) treatments (mean ± SD, *n* = 3). One-way ANOVA and Duncan’s multiple range test (*p* < 0.05). Different letters indicate significant differences.

The expression profiles of *MsPILSs* after ABA therapy are illustrated (Figure 8A). *MsPILS3c*, *MsPILS11a*, and *MsPILS16d* demonstrated significant overexpression, while other genes displayed downregulation. The expression of *MsPILS11a* progressively elevated with time, attaining its peak at 48 h. *MsPILS3c* exhibited substantial upregulation at 3 h, followed by a slow decline over time.

The expression profiles of *MsPILSs* following SA treatment are illustrated (Figure 8B). The relative expression of *MsPILS1c* and *MsPILS3b* was significantly elevated at 6 h, achieving 71-fold and 51-fold increases compared to the control, respectively, before gradually declining. *MsPILS4a* transcript levels were significantly elevated at 12 h and thereafter diminished progressively. The expression of *MsPILS16d* progressively rose from 6 h to 24 h, reaching its zenith at 24 h.

The expression profiles of *MsPILSs* following GA treatment are illustrated (Figure 8C). During the early period from 3 to 12 h, the majority of *MsPILS* genes showed significant downregulation. *MsPILS1b* diminished by almost 50-fold at 3 h. Most other genes exhibited a drop of 7- to 100-fold, with the exception of *MsPILS11a*, which demonstrated the most significant decrease of 476-fold at 6 h. The expression levels of *MsPILS1c* and *MsPILS3b* reached their zenith at 6 h, then declined, and then increased again at 48 h. *MsPILS1b*, *MsPILS3c*, *MsPILS11a*, and *MsPILS15b* exhibited elevated expression levels at 24 h. In comparison to the control, *MsPILS3c* had the greatest expression of all genes at both 24 h and 48 h.

The *MsPILS* gene family demonstrates varied expression patterns in response to ABA, SA, and GA treatments, indicating their incorporation into separate phytohormone signaling networks. The upregulation of some members in response to ABA and SA indicates their role in stress adaptation and defense signaling, whereas the temporal cascade associated with GA demonstrates stringent transcriptional regulation. These findings identify *MsPILS* genes as possible regulatory centers coordinating hormone-mediated adaptation responses in alfalfa.

### 3.11. Expression Profiles of MsPILS Genes in Diverse Tissues

**Figure 9:**
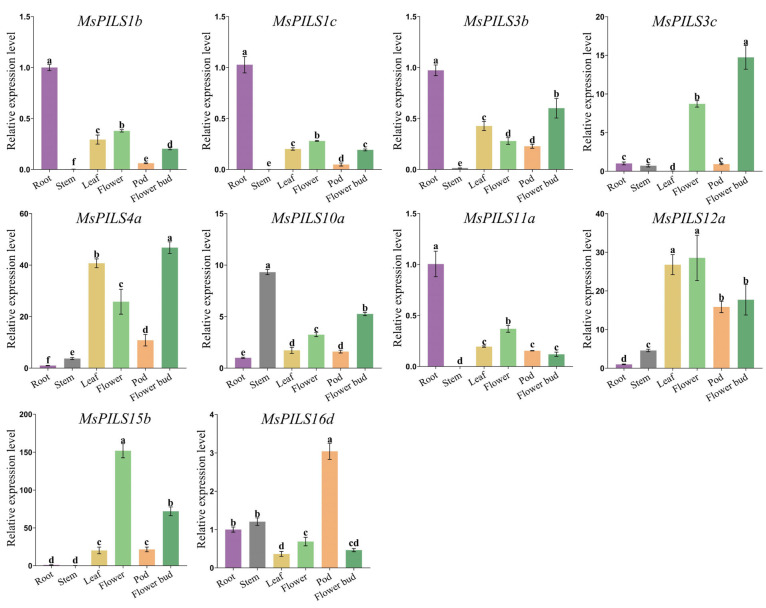
Expression patterns of *PILS* gene family members in different tissues of *M. sativa* are shown as mean ± SD (*n* = 3). One-way ANOVA followed by Duncan’s test (*p* < 0.05). Different lowercase letters indicate significant differences.

The expression levels of 10 *MsPILS* genes were examined in six distinct tissues (root, stem, leaf, flower, flower bud, and pod) of *M. sativa* (Figure 9). Specifically, *MsPILS1b* and *MsPILS1c* exhibited elevated expression in roots, with transcript levels roughly 200- and 600-fold greater than those in stems, respectively, indicating their possible pivotal roles in root morphogenesis or auxin signaling. Conversely, *MsPILS12a* and *MsPILS15b* exhibited predominant expressions in flowers, with levels markedly exceeding those in roots, suggesting their role in floral organ development or the regulation of flowering time. A handful of genes, including *MsPILS10a* and *MsPILS12a*, exhibited markedly elevated expression in stems compared to roots. Although the majority of genes exhibited moderate expression in leaves, only *MsPILS1b*, *MsPILS3b*, and *MsPILS4a* were expressed at significantly elevated levels in leaves compared to blossoms. Collectively, these findings indicate distinct functional diversity within the *MsPILS* family. The delineated expression patterns offer essential insights for forthcoming functional investigations and for pinpointing potential genes pertinent to the enhancement of root architecture and blooming characteristics in *M. sativa*.

### 3.12. Subcellular Localization of MsPILS1c Protein

**Figure 10:**
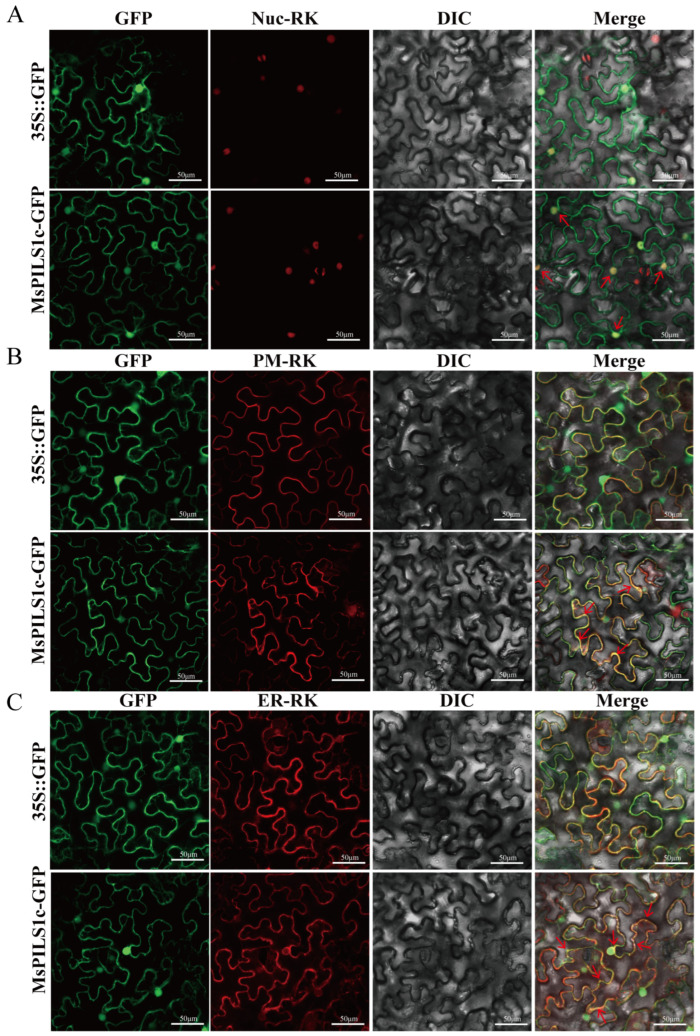
Subcellular localization of the MsPILS1c protein. Figures (**A**–**C**) show MsPILS1c-GFP co-expressed with a free GFP control (35S::GFP) and with a nuclear marker (**A**), a plasma membrane marker (**B**), or an endoplasmic reticulum marker (**C**), respectively. Green: GFP; Red: organelle marker; DIC: dark-field; Merge: merged image. Nuc-RK: mCherry-tagged nuclear marker; PM-RK: mCherry-tagged plasma membrane marker; ER-RK: mCherry-tagged endoplasmic reticulum marker. Scale bar = 50 μm.

MsPILS1c was chosen for the subcellular localization study due to its significant differential expression under PEG, NaCl stress, and following SA and GA treatments. A transient expression test of the pCAMBIA2300-MsPILS1c-GFP fusion protein was performed in *Nicotiana benthamiana* leaves to ascertain the subcellular location of the MsPILS1c protein (Figure 10). Confocal laser scanning imaging demonstrated that free GFP in the control group (35S::GFP) displayed diffuse fluorescence throughout the cells and did not colocalize with any organelle markers, signifying its lack of enrichment in specific subcellular structures. Red arrows are used in Figure 10 to highlight the differences between MsPILS1c-GFP and 35S::GFP. MsPILS1c-GFP demonstrated specific localization, colocalizing with the nuclear marker (Nuc-RK) (Figure 10A), the plasma membrane marker (PM-RK) (Figure 10B), and the endoplasmic reticulum marker (ER-RK) (Figure 10C). The results indicate that MsPILS1c is localized in the nucleus, plasma membrane, and ER.

## 4. Discussion

The *PILS* gene family is a crucial regulator of plant auxin equilibrium and stress adaptation. Its functions are chiefly linked to the control of nuclear auxin signaling, responses to abiotic stress, and organ development. Nonetheless, the *PILS* gene family in *M. sativa*, a significant leguminous fodder crop, has not been comprehensively characterized. This gap has constrained a comprehensive understanding of the auxin regulatory network and the molecular processes that govern stress tolerance in *M. sativa*. This work discovered 46 *MsPILS* genes based on the genomic data of tetraploid *M. sativa*. Utilizing extensive bioinformatic analysis and qRT-PCR validation, we systematically clarified their evolutionary relationships, chromosomal distribution, expression patterns, and subcellular localization, therefore supplying critical data to bridge this research gap.

The number of *PILS* genes varies significantly among different plants: for instance, 7 in *A. thaliana*, 13 in the rubber tree [19], and 13 in *Chrysanthemum seticuspe* [18]. In contrast, alfalfa possesses 46 *MsPILS* genes, a number substantially higher than in the other species examined. This expansion may be related to the tetraploid nature of the alfalfa genome. Differences in *PILS* gene copy numbers across plant species can generally be attributed to gene duplication events, variations in genome size, and gene loss during evolution [44]. Gene duplication events, including whole-genome duplication (WGD) [45], tandem duplication [46], and segmental duplication [47], are central drivers of plant gene family evolution [48]. The calculated Ka/Ks ratios for most *MsPILS* gene pairs were less than 1, indicating that these genes have predominantly undergone purifying selection during evolution. Furthermore, we identified 29 segmentally duplicated *MsPILS* gene pairs, suggesting that segmental duplication has been a significant mechanism for the expansion of this gene family in *M. sativa*.

A phylogenetic study categorized the *M. sativa* PILS family into four major clades (Figure 1). In contrast to the rubber tree, which has just two identified clades [19], the greater number of clades in alfalfa may indicate lineage-specific gene duplication and retention events during legume evolution. The variation in motif compositions among clades underscores the universal conservation of motif 2, emphasizing its essential role in the basic transport function of PILS proteins. The prevalent lack of motif 10 suggests that this element may have become superfluous or functionally substituted during the evolution of *PILS* genes in *M. sativa*. The markedly increased number of exons in Clade B members indicates that genes within this clade may exhibit enhanced complexity at the transcriptional level or via alternative splicing. The clade-specific structural characteristics suggest that the four evolutionary clades of *M. sativa PILS* genes are not mere accumulations of gene duplicates but rather likely signify separate evolutionary units that have experienced the first functional differentiation.

MsPILS proteins predominantly comprise *α*-helices that constitute the structural core, exhibiting subfamily-specific differences in the number and length of helices. The PPI network prediction indicated that MsPILS10a/b/c/d interact with NADP-malic enzyme (NADP-ME) proteins, which play a role in stress responses. MeNADP-ME3 has demonstrated the ability to enhance salt and drought tolerance in Arabidopsis [49]. Numerous MsPILS proteins were identified to interact with PIN3, PIN5, and PIN6, which are critical auxin efflux carriers vital for plant growth and development [50,51]. The interactions indicate that PILS proteins may operate at the junction of auxin homeostasis and stress adaptation in *M. sativa*.

Analysis of cis-acting elements in the promoter regions indicated a prevalence of light-responsive, hormone-responsive, and stress-responsive elements in the alfalfa *PILS* gene family. This suggests their potential extensive role in photosynthesis, hormone signaling, and stress response mechanisms in alfalfa. The widespread occurrence of light-responsive regions in all *MsPILS* genes indicates a conserved potential function in light-mediated regulation. Markers such as ARE and MBS, indicative of abiotic stress induction, were discovered, suggesting a role for *MsPILS* genes in reacting to stimuli such as elevated salinity, low temperature, and drought. Multiple hormone-responsive elements associated with salicylic acid (SA), gibberellins (GA), auxin, and abscisic acid (ABA) were identified.

In contrast to migratory species, plants are stationary and must depend on internal regulating mechanisms to withstand unfavorable climatic conditions [52,53]. In response to stress, auxin transporters regulate adaptive cellular and organ-level responses by altering the spatiotemporal distribution of auxin, therefore improving plant stress adaptation [54,55,56]. Under abiotic stress conditions, members of the *MsPILS* family have varied expression patterns. Particularly, many members of gene families are induced by low temperature, drought, and salinity stress. The findings align with previous reports: *CsPILS* genes in Chrysanthemum seticuspe are involved in drought stress response [18], while *HbPILS6* in rubber tree exhibits significant expression alterations under salt and drought stress [19]. In situations of salt stress, the expression of *LiPILS* genes in *Lagerstroemia indica* was markedly increased [20]. This validates the conserved function of the *PILS* gene family in plant response to abiotic stressors. Under PEG-induced drought stress, the majority of *MsPILS* genes exhibited a distinctive expression fluctuation pattern characterized by an “increase-decrease-increase-decrease” dynamic. The timing of these peaks coincided with the dark period at 12 h and 48 h following the commencement of treatment. This species-specific pattern may be associated with a distinct mechanism in alfalfa, wherein auxin homeostasis is modulated by light cues to enhance drought adaptation, providing a fresh framework for the comprehensive study of stress adaptation techniques in forage crops.

Auxin, as a crucial regulator of plant physiology and stress tolerance, orchestrates its functions through hormonal interactions, especially with ABA, SA, and GA, to precisely regulate its homeostasis and signaling responses [57,58,59,60]. The *MsPILS* genes exhibit significant specificity and interaction in their responses to hormonal cues. Some family members are governed by a singular hormone, whereas others react to various hormonal signals. This corresponds with established regulation patterns, shown by the *TGA7*-*PILS2* module in tomato, which integrates hormonal inputs to modulate plant development [34], and the *PILS* genes in *Chrysanthemum seticuspe* that respond to ABA and SA signals during stress responses [18]. This indicates that *MsPILS* genes may jointly govern alfalfa’s environmental adaptability by combining hormonal signaling and stress response pathways, thus enhancing the theoretical framework of the synergistic regulatory network between plant hormones and auxin transporters.

The transcriptional responsiveness of *PILS* members was associated with the type and number of cis-acting elements in their promoters. For instance, *MsPILS3c* and *MsPILS14a* had several ABREs and exhibited significant upregulation in response to ABA. These data align with the recognized notion that composite cis-element modules, rather than discrete motifs or mere copy number, regulate transcriptional responses to environmental stimuli [61]. The combinatorial arrangement of cis-elements seems to establish the transcriptional identity of *MsPILS* genes in response to stress and hormonal therapies.

Analysis of tissue-specific expression demonstrated functional differences among *MsPILS* genes. *MsPILS1b* and *MsPILS1c* exhibited elevated expression levels in root tissue. This outcome aligns with data indicating that *CsPIN3* in *Areca catechu* is predominantly expressed in roots and governs adventitious root development, while *AcPIN6* is exclusively expressed in prop roots and contributes to root formation [33]. This suggests that *MsPILS1b* and *MsPILS1c* may serve a fundamental regulatory function in root morphogenesis, the maintenance of auxin homeostasis, and root stress tolerance in alfalfa. Specifically, *MsPILS6* and *MsPILS10a* had an inverse expression pattern, with their expression levels in pods being inferior to those in stems. This distinctive pattern diverges from the prevailing expression of the majority of *PILS* genes in pods, indicating that the primary roles of *MsPILS6* and *MsPILS10a* pertain to stem development, stem elongation, or auxin transport during vegetative growth, rather than reproductive development. *MsPILS6* and *MsPILS15b* demonstrated exceptional expression selectivity in floral tissue. The identification of *PILS* genes in *Chrysanthemum seticuspe*, which are implicated in flower bud differentiation and floral organ development [18], further substantiates the hypothesis that *MsPILS6* and *MsPILS15b* may be crucial in regulating blooming time, petal development, or pollen generation in alfalfa.

Subcellular localization is essential for protein functionality. Proteins in the endoplasmic reticulum or vacuoles typically facilitate autophagy and transport, whereas nuclear proteins predominantly govern transcriptional regulation [42]. MsPILS1c localizes to the plasma membrane, according to bioinformatic prediction, and subcellular localization studies showed that it localizes to the nucleus, plasma membrane, and ER. This dual localization is very different from both the exclusive plasma membrane localization of HbPILS6 in rubber trees [19] and the predominant ER localization of most PILS proteins in *A. thaliana* [6]. This threefold localization indicates the several functional states of MsPILS1c: it participates in auxin efflux at the cell membrane, is generated and stored in the endoplasmic reticulum, and may execute regulatory roles within the nucleus that remain unexplained. The mechanism by which this protein enters the nucleus is an area requiring further exploration; it may entail a concealed nuclear localization signal, interactions with nuclear transport receptors, or the formation of a soluble nuclear variant via post-translational modifications under particular conditions. While these possibilities necessitate more validation, the identification of this multi-localization offers significant insights for a more profound comprehension of the potential functions of MsPILS1c beyond traditional auxin transport.

## 5. Conclusions

In this study, we performed the first genome-wide identification and characterization of the *PILS* gene family in tetraploid alfalfa. We comprehensively analyzed the bioinformatic characteristics of the *MsPILS* gene family, including their gene structures, chromosomal distributions, phylogenetic relationships, conserved motifs, cis-elements, and PPI networks. Furthermore, expression profiling revealed that *MsPILS* genes exhibit tissue-specific patterns and are differentially regulated under cold, drought, and salt stresses, suggesting their functional diversification in development and stress adaptation. Subcellular localization investigations revealed that MsPILS1c is situated in the nucleus, plasma membrane, and ER. Overall, this study provides key genetic resources for functional characterization of the *PILS* family in alfalfa and establishes a foundation for breeding programs aimed at enhancing stress tolerance.

## Data Availability

The original contributions presented in this study are included in the article/Supplementary Materials. Further inquiries can be directed to the corresponding author.

## Supplementary Materials

The following supporting information can be downloaded at: https://www.mdpi.com/article/10.3390/cimb48060580/s1.
