# Supplementary material for: Genome-Wide Identification of the PIN-LIKES (PILS) Gene Family in Alfalfa (Medicago sativa L.) and Its Expression Analysis Under Abiotic Stresses
Source: Curr Issues Mol Biol. 2026 Jun 1;48(6):580. doi: 10.3390/cimb48060580 (PMC13297628; doi:10.3390/cimb48060580)
Supplement: Supplementary file 1 [file cimb-48-00580-s001.zip › cimb-4260952-supplementary.pdf]

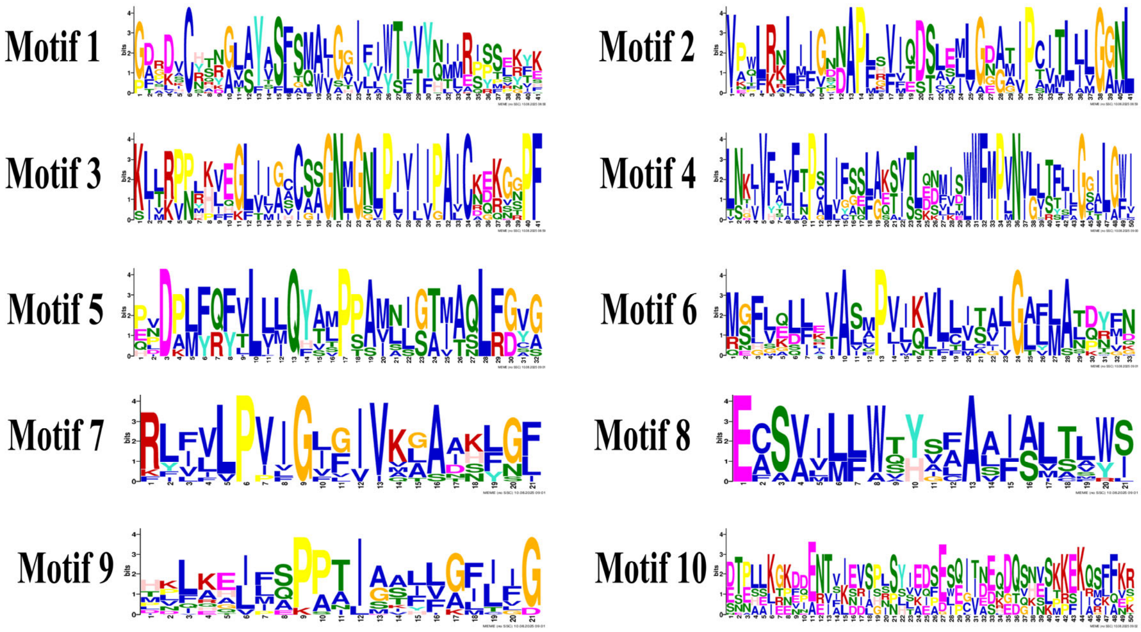

FigureS1. Sequence logos for motif1-10. Each conserved motif is represented as a sequence logo, where the amino acid sequence is depicted by stacked letters at each position.

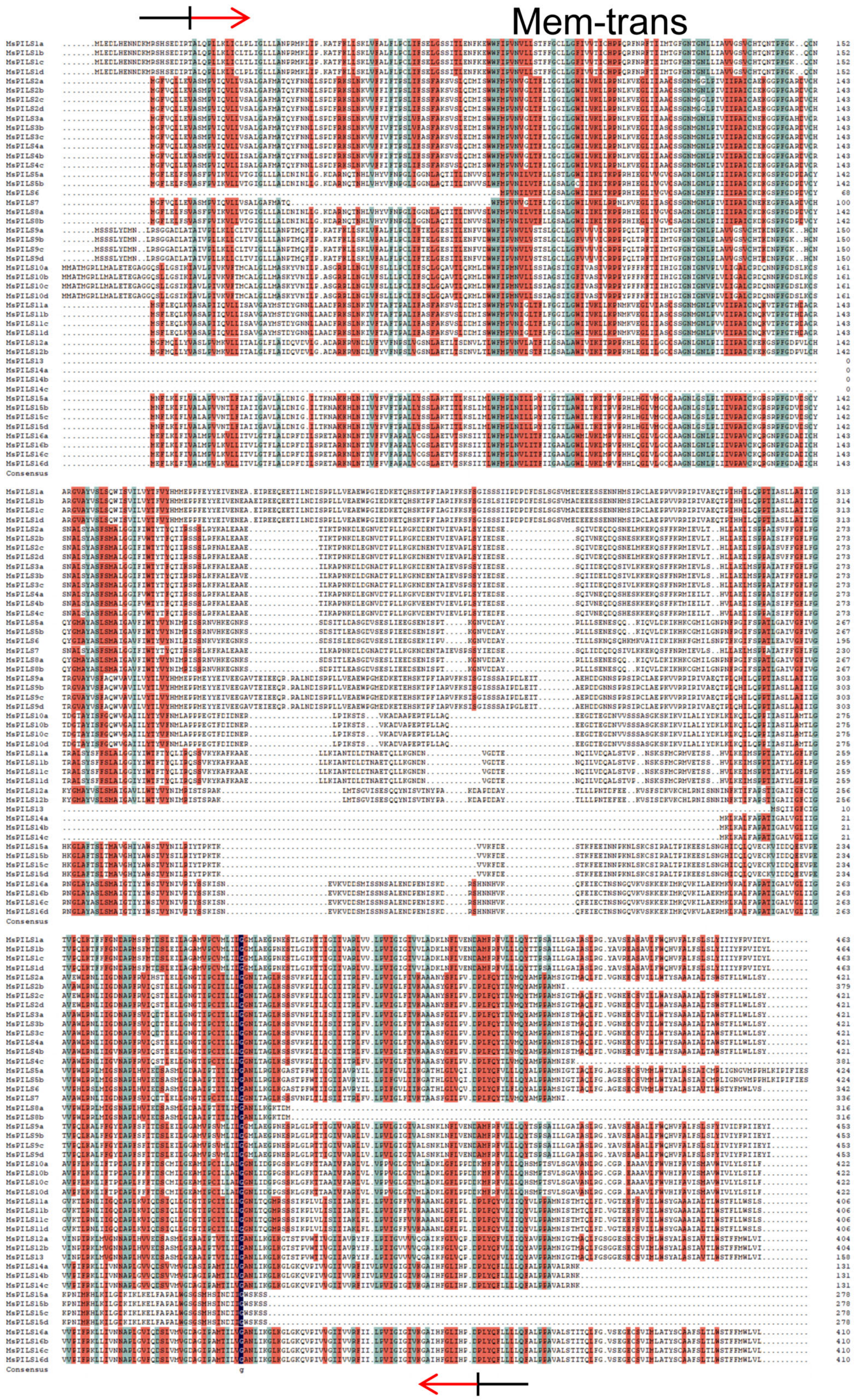

Figure S2. Multiple sequence alignments of the conserved domains of the MsPILS proteins. The domain names are shown at the top of the sequence. Protein sequence names are shown on the left. The conserved amino acid residues are shaded in color.

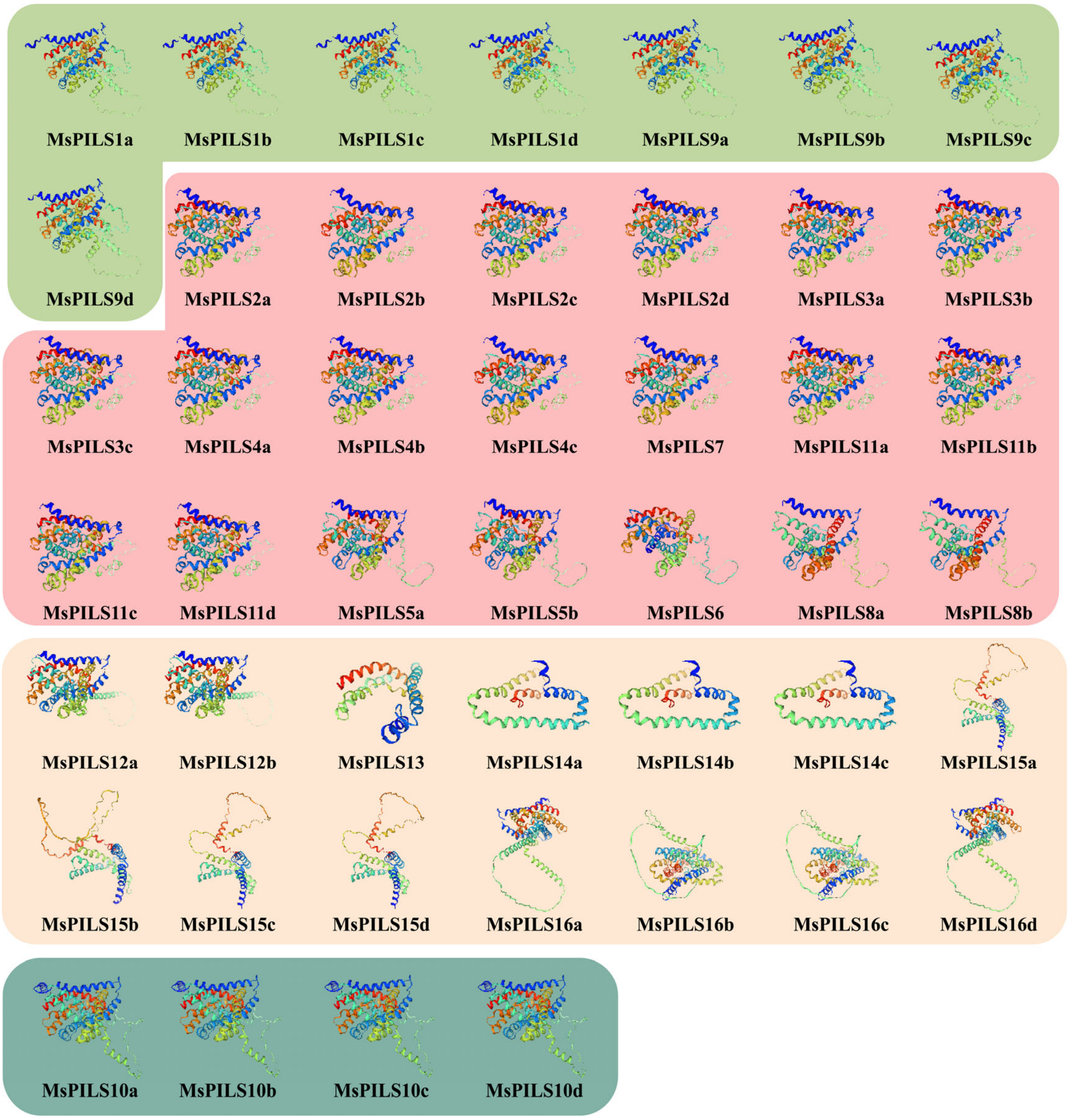

Figure S3. Predicted tertiary structure of MsPILS proteins.
